# *Eisenia andrei* and *Tenebrio molitor* Divergently Restructure the Bacteriome and Mycobiome of Sewage Sludge with Contrasting Biosafety Consequences

**DOI:** 10.3390/biotech15030062

**Published:** 2026-08-03

**Authors:** Eduardo Mancilla, Marcos Pérez-Losada, Manuel Aira, Jorge Domínguez

**Affiliations:** 1 Department of Biostatistics and Bioinformatics, Computational Biology Institute, Milken Institute School of Public Health, George Washington University, Washington, DC 20052, USA; jemancilla@gwu.edu (E.M.); mlosada@gwu.edu (M.P.-L.); 2 Grupo de Ecoloxía Animal (GEA), Universidade de Vigo, 36310 Vigo, Spain; jdguez@uvigo.gal

**Keywords:** *16S*, bacteria, fungi, *ITS*, earthworms, human pathogen, microbiome, sewage sludge

## Abstract

The use of invertebrates for sewage sludge bioconversion offers a sustainable strategy for waste valorization, yet species-specific effects on microbial communities and biosafety remain unclear. Here, we compared the impacts of the earthworm *Eisenia andrei* (Ea) and the mealworm *Tenebrio molitor* (Tm) on the bacteriomes and mycobiomes of sewage sludge (ss) using *16S rRNA* and *ITS* amplicon sequencing. Gut passage in both Ea and Tm markedly altered bacterial and fungal composition relative to ss, but produced distinct community profiles with differential shifts across multiple taxa. Both invertebrates reduced bacterial richness by ~40%, while fungal responses diverged: Ea largely preserved mycobiome richness despite reduced evenness, whereas Tm caused a near-complete collapse (~83% Amplicon Sequence Variant loss). Beta diversity analyses revealed clear, non-overlapping separation among ss, Ea, and Tm for both microbial domains. Tm frass showed strong enrichment of clinically relevant bacterial pathogens, while Ea casts exhibited no such enrichment. For fungi, Ea reshaped pathogen composition, whereas Tm largely eliminated fungal pathogens through broad community collapse. Both treatments reduced predicted antibiotic resistance gene abundance, but functional profiles differed, with Ea showing greater functional stability. These findings demonstrate that microbiome restructuring during bioconversion is species-dependent, with contrasting ecological and biosafety implications for downstream environmental use.

## 1. Introduction

Beyond its organic load and heavy metal content, sewage sludge harbors a compositionally complex microbial community that routinely includes human bacterial pathogens, antibiotic resistance genes (ARGs), and opportunistic fungal hazards that conventional stabilization methods reduce only partially [1]. Global sludge production continues to rise in step with urbanization, with European Union countries generating more than 10 million tons of dry matter annually [2], placing increasing pressure on downstream management strategies to reliably reduce pathogen and resistance risks without the energy and cost burdens of thermophilic digestion or thermal drying. The persistence of *Enterococcus* spp., *Pseudomonas aeruginosa*, *Salmonella*, and Enterobacteriaceae in treated sludge poses direct risks when sludge is applied to agricultural land [1]. Moreover, sludge is increasingly recognized as a hotspot for ARG dissemination into food chains and natural ecosystems [3].

Conventional sludge treatment methods, including thermophilic anaerobic digestion, lime stabilization, and thermal drying, can partially reduce pathogen loads but remain energy-intensive, costly, and often incomplete. Biological approaches that harness the digestive activity of invertebrates have attracted growing scientific interest as potentially resource-efficient and ecologically sustainable alternatives. Vermicomposting, the processing of organic waste by epigeic earthworms, has been shown to substantially reshape microbial community composition, reduce pathogen abundance, and enhance the functional potential of the resulting material [4]. *Eisenia andrei* (Bouché 1972), one of the most extensively studied vermicomposting species, imposes large-scale restructuring of both the bacteriome and mycobiome of sewage sludge through the specific physicochemical conditions of its gut, including changes in pH, enzymatic activity, mucus secretions, and transit time that collectively act as a selective ecological filter on the ingested microbial community [5].

Insect-mediated bioconversion of organic waste has more recently emerged as an alternative or complementary strategy for valorizing low-quality substrates. The yellow mealworm *Tenebrio molitor* (Linnaeus, 1758) is among the most widely studied insect species for this purpose, primarily in the context of food waste and agricultural wastes [6]. *T. molitor* processes ingested substrate through a gut physiology that differs substantially from that of earthworms, featuring a distinct gut pH profile, shorter transit time, a characteristic Bacillota-enriched larval gut microbiome, and production of frass that is compositionally and chemically distinct from earthworm casts [7,8]. Despite growing interest in *T. molitor* as a bioconversion agent, the effects of mealworm gut transit on the microbial communities of sewage sludge have not been fully characterized, and a rigorous comparison with earthworm processing under identical experimental conditions has not yet been performed. Such a study would enable a comprehensive evaluation of the advantages and limitations of these invertebrate bioconversion systems, as well as their ecological and biosafety implications for sewage sludge valorization.

Here we directly compare the bacteriome and mycobiome of fresh sewage sludge with those of *E. andrei* casts and *T. molitor* frass produced from the same substrate batch under identical experimental conditions. Our central question is whether these two invertebrate species impose similar or divergent effects on microbial community composition, diversity, and structure, predicted functional potential, and pathogen dynamics, and what these outcomes imply for the downstream regulation of invertebrate-processed sludge.

## 2. Materials and Methods

### 2.1. Sample Collection

Fresh sewage sludge was collected from a municipal wastewater treatment plant immediately after the dewatering stage. Five aliquots of sewage sludge (ss) were homogenized and stored at −80 °C until DNA extraction. Sewage sludge used in this experiment was characterized by a slightly basic pH (8.77 ± 0.02), high electric conductivity (892 ± 11 µs cm^−1^), high moisture (83.5 ± 0.1%), and relatively high organic matter content (68.4 ± 0.3%). Separate aliquots of fresh sludge were simultaneously provided ad libitum to *T. molitor* larvae (Tm) and *E. andrei* earthworms (Ea) under controlled laboratory conditions: 20 °C and 80% and 70% relative humidity for Ea and Tm, respectively. Specimens were kept in identical containers of 4 L in volume. Fresh sludge was provided as feed to ongoing laboratory cultures of each invertebrate species already feeding on sewage sludge from the same wastewater treatment plant. All cultures were maintained under the same conditions and processed identically. After 48 h of feeding, we collected 5–20 individuals per Ea and Tm species from each culture. A 48 h interval is considered sufficient for ingested material to pass through the earthworm and mealworm gut and produce new dejections [5,9]. *E. andrei* earthworms were sequentially washed with tap and sterile water, placed in five sterile Petri dishes, and incubated for 24 h to allow gut voiding, after which freshly deposited cast samples were aseptically collected using a sterile spatula. *T. molitor* frass was collected by picking larvae and leaving them to void their guts in seven sterile Petri dishes directly following the same 48 h feeding period. Each Petri dish represents a composite sample of feces from several earthworms or insect larvae. All samples were stored at −80 °C in Eppendorf tubes until further processing. The total dataset comprised 17 samples: 5 ss, 5 Ea casts, and 7 Tm frass.

### 2.2. DNA Extraction, Amplification, and Sequencing

Total DNA was extracted from approximately 0.25 g of fresh weight of each sample in a laminar flow cabinet using the MO BIO PowerSoil DNA Isolation Kit (Qiagen, Hilden, Germany) following the manufacturer’s protocol. The V4 hypervariable region of the *16S* rRNA gene was amplified for bacterial community characterization, and the *ITS1* region was amplified for fungal community analysis. Both amplicons were sequenced on an Illumina Novaseq platform at Novogene, Cambridge, UK using paired-end 2 × 250 bp chemistry. Negative controls were included during DNA extraction and showed no PCR amplification. Subsequent sequencing of these controls yielded no reads.

### 2.3. Bioinformatic and Statistical Analysis

Amplicon sequence variants (ASVs) were inferred using DADA2 (v1.34.0) [10] under its standard paired-end workflow. Raw reads were trimmed based on per-base quality scores, then processed through error modeling, dereplication, denoising, paired-end merging, and chimera removal. Taxonomic assignment of *16S* ASVs was performed against the SILVA database (version 138.2) [11] using the Bayesian classifier [12], with species-level annotation further refined using the DADA2 assignSpecies function. *ITS* ASV taxonomy was assigned against the UNITE database (version 10) [13]. ASVs represented by a single read across all samples (singletons) were removed prior to all analyses; the resulting dataset retained at least two reads per ASV across all samples. Sequence data were deposited in the GenBank SRA database under accession numbers PRJNA1479214 and PRJNA1479220 for the bacteriome and mycobiome, respectively.

All analyses were conducted in R v4.6.0 and RStudio v2026.4.0.526 using the packages phyloseq v1.52.0 [14], tidyverse v2.0.0 [15], vegan v2.7-5 [16], rstatix v0.7.3 [17], microeco v1.16.0 [18], and DESeq2 v1.48.1 [19]. Before differential abundance testing, ASVs present in fewer than 10% of samples (prevalence threshold = 0.10; equivalent to presence in at least 2 of 17 samples) were removed. For the bacteriome, this threshold excluded 41% of ASVs, representing approximately 0.63% of total sequences. For the mycobiome, 63% of ASVs were excluded, representing approximately 1.11% of total sequences. Differential abundance between treatment groups was estimated using DESeq2 at the phylum and genus levels. The model applied a Wald test with parametric dispersion fitting; size factors were estimated using the positive count geometric mean approach to address zero inflation. For the bacteriome, mycobiome, and human bacterial pathogen (HBP) analyses, sewage sludge (ss) was set as the reference group, yielding comparisons of Ea vs. ss and Tm vs. ss. For the HBP analysis, the factor level order was explicitly set so that ss remained the reference (levels: ss, Ea, Tm). Results were considered statistically significant at a Benjamini–Hochberg-adjusted *p*-value (*P*~adj~) ≤ 0.05.

Alpha diversity was estimated from rarefied subsamples using repeated rarefaction to the minimum sample sum (b = 100 iterations, fixed random seed), averaging across iterations to reduce stochastic variance. Four indices were calculated for the bacteriome, namely, ASV richness (Observed), Shannon diversity, inverse Simpson diversity, and Faith’s phylogenetic diversity (FaithPD; calculated using the picante package v1.8.2), while three indices were calculated for the mycobiome (Observed, Shannon, and inverse Simpson). Alpha diversity indices were compared using Kruskal–Wallis tests followed by pairwise Wilcoxon post hoc tests with FDR correction, given the failure of the normality assumptions as confirmed by Levene and Shapiro–Wilk tests.

Beta diversity was analyzed on variance-stabilizing transformed (VST) count matrices generated using DESeq2. Principal coordinates analysis (PCoA) was performed on Bray–Curtis and Jaccard distances. Group level differences were evaluated using PERMANOVA (vegan package v2.7.3; adonis2) at both overall and pairwise levels, with pairwise *p* values adjusted using FDR correction.

### 2.4. Human Pathogen Analysis

Human bacterial pathogens (HBPs) were identified by cross-referencing the full species-level taxonomy table against a comprehensive curated list of bacterial species with established or putative human pathogenic status [20], returning 29 HBP species across the dataset. Differentially abundant HBPs were identified using DESeq2 with ss set as the reference group. Human fungal pathogens (HFPs) were screened by matching ITS-derived taxonomy against the WHO Fungal Priority Pathogens List [21] and VEuPathDB [22].

### 2.5. Bacterial Functional Analysis

Bacterial functional profiles were inferred with KEGG Orthology (KO) abundances derived from rarefied 16S ASV data [23]. Prediction confidence was assessed using the Nearest Sequenced Taxon Index (mean weighted NSTI = 0.048 ± 0.001), well below the recommended threshold of 0.1, indicating high predictive accuracy across all samples. Beta diversity analyses of functional profiles were carried out as indicated above for taxonomic distances and tested via PERMANOVA. Differences in KO abundances across seven functional categories (antibiotic resistance, antibiotic biosynthesis, amino acid biosynthesis, bisphenol degradation, furfural degradation, nitrogen metabolism, and plant hormone synthesis) were assessed using pairwise Wilcoxon tests with FDR correction, following confirmation that parametric ANOVA assumptions were violated (Levene test; normality check). KEGG pathway-to-KO mappings were retrieved using KEGGREST version 1.1 [24].

### 2.6. Fungal Functional Guild Analysis

Fungal ecological guilds were assigned using FUNGuildR (version 0.3.0) [25], a tool that maps ASV taxonomy to a curated ecological trait database. Guild assignments were restricted to ASVs rated as Probable or Highly Probable confidence levels to minimize false positive trait assignments. Trophic modes were classified into 7 categories, including Saprotroph, Pathotroph, Symbiotroph, and mixed-mode designations. Differences in trophic mode richness and abundance among ss, Ea, and Tm were assessed using Kruskal–Wallis tests, followed by pairwise Wilcoxon post hoc tests with FDR correction. ASVs that did not receive a guild assignment were retained in compositional analyses but excluded from guild-specific comparisons.

All datasets were assessed for compliance with the assumptions of the applied statistical methods and were found suitable for DESeq2, Kruskal–Wallis, and PERMANOVA analyses.

## 3. Results

We analyzed 5 samples of fresh sewage sludge (ss), 5 samples of *E. andrei* casts (Ea), and 7 samples of *T. molitor* frass (Tm). Rarefaction curves based on the full bacteriome and mycobiome datasets, with 1000 sequences at each step, showed that all 17 samples had reached saturation. This confirmed that the sequencing depth was sufficient for a robust characterization of all bacteriomes and mycobiomes (Supplementary Figure S1). UpSet plot analysis revealed that the large majority of ASVs were unique to individual treatment groups in the bacteriome (ss: 447; Ea: 437; Tm: 842), with limited sharing between pairs (ss–Ea: 130; ss–Tm: 205; Ea–Tm: 82) and only 242 ASVs shared across all three groups (Supplementary Figure S2). A similar pattern was observed for the mycobiome, where most ASVs were group-specific (ss: 619; Ea: 694; Tm: 58), with modest pairwise overlap (ss–Ea: 332; ss–Tm: 15; Ea–Tm: 19) and 83 ASVs shared across all three groups (Supplementary Figure S2).

### 3.1. Bacteriome Composition, Diversity, Structure, and Differential Abundance

**Figure 1:**
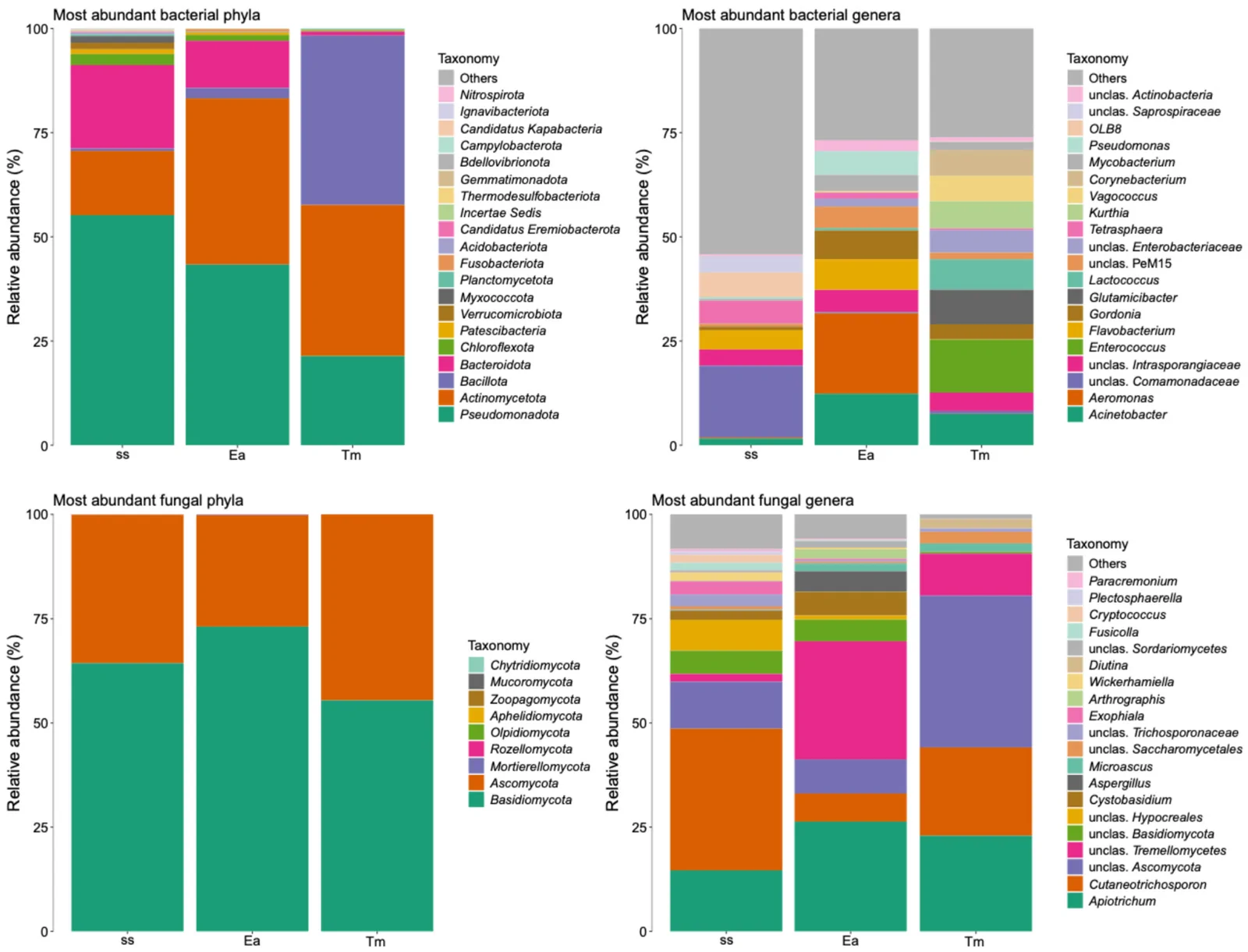
Relative mean abundance of the most abundant phyla and genera in the bacteriomes and mycobiomes of sewage sludge (ss), *Eisenia andrei* casts (Ea), and *Tenebrio molitor* frass (Tm).

Bacteriome composition differed markedly among the three groups at both the phylum and genus levels (Figure 1 and Supplementary Table S1). At the phylum level, the ss bacteriome was dominated by Pseudomonadota and Actinomycetota, with minor contributions from Bacillota, Bacteroidota, and Chloroflexota. The Ea bacteriome was codominated by Pseudomonadota and Actinomycetota, with Bacteroidota and Bacillota present as secondary phyla. The Tm bacteriome differed substantially, being predominantly composed of Bacillota and Actinomycetota, with Pseudomonadota representing a comparatively reduced fraction. At the genus level, the ss bacteriome was characterized by unclassified Comamonadaceae (17.2 ± 0.8%), OLB8 (5.9 ± 0.1%), *Tetrasphaera* (5.5 ± 0.6%), *Flavobacterium* (4.7 ± 0.3%), and unclassified Intrasporangiaceae (3.9 ± 0.4%) as the most abundant taxa. The Ea bacteriome was dominated by *Aeromonas* (19.3 ± 1.1%), *Acinetobacter* (12.4 ± 3.4%), *Flavobacterium* (7.4 ± 0.6%), *Gordonia* (6.9 ± 0.5%), *Pseudomonas* (5.7 ± 0.6%), and unclassified PeM15 (5.1 ± 0.4%). The Tm bacteriome was characterized by *Enterococcus* (12.6 ± 1.6%), *Glutamicibacter* (8.3 ± 1.0%), *Acinetobacter* (7.5 ± 1.6%), *Lactococcus* (7.4 ± 1.9%), *Kurthia* (6.5 ± 1.2%), *Corynebacterium* (6.2 ± 0.6%), and *Vagococcus* (6.0 ± 1.1%) as dominant genera.

A total of 17 bacterial phyla showed significant differences in abundance between ss and Ea, 15 between ss and Tm, and 14 between Tm and Ea (all *P*adj ≤ 0.046; Supplementary Table S2). At the genus level, 274 genera differed significantly between ss and Ea, 264 between ss and Tm and 185 between Tm and Ea (all *P*adj ≤ 0.048; Supplementary Table S2).

**Figure 2:**
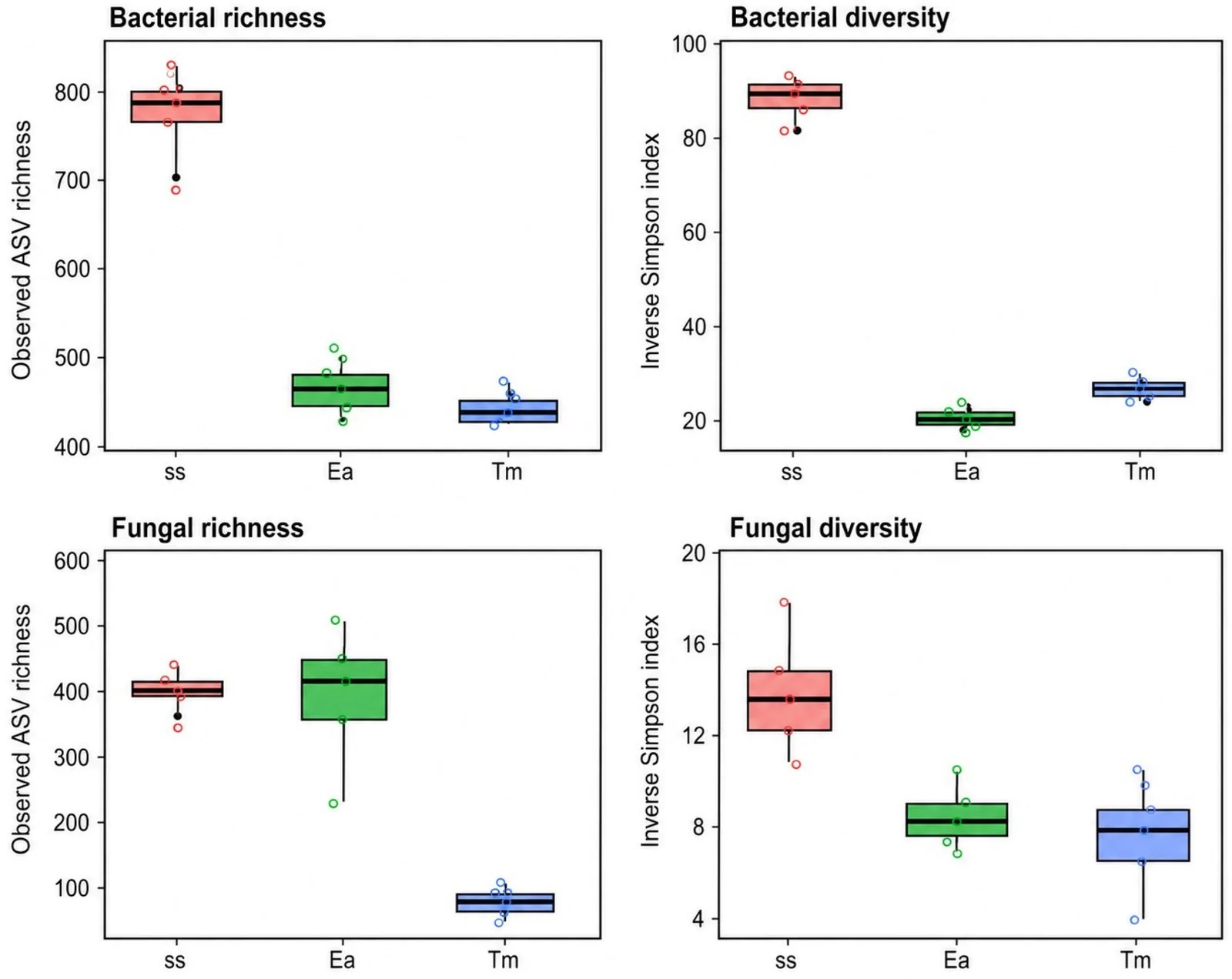
Box plots of alpha diversity estimates for bacteriomes and mycobiomes of sewage sludge (ss), *Eisenia andrei* casts (Ea), and *Tenebrio molitor* frass (Tm). The small circles represent individual samples (replicates). The box spans the interquartile range and the thick horizontal line in the middle represents the median estimate.

Bacteriome alpha diversity was significantly lower in both Ea and Tm relative to ss across all metrics (Figure 2; pairwise Wilcoxon, all *P*adj ≤ 0.048), apart from Observed ASV richness, which did not differ significantly between Ea and Tm (*P*adj = 0.745). Sewage sludge harbored the highest species richness, and invertebrate digestion significantly reduced it (−39% and −40% in Ea casts and Tm frass respectively; Figure 2). Inverse Simpson followed the same trend with significant reductions in Ea casts (−77%) and Tm frass (−70%; Figure 2). Shannon diversity also declined from 5.365 ± 0.019 in ss to 4.294 ± 0.058 in Ea and 4.254 ± 0.022 in Tm, as it occurred with Faith’s phylogenetic diversity (ss: 58.2 ± 1.5; Ea: 32.6 ± 0.7; Tm: 30.3 ± 0.5). Ea casts and Tm frass did not differ significantly in observed ASV richness and Shannon diversity; however, Inverse Simpson and Faith’s PD were significantly higher in Tm than in Ea (*P*adj = 0.010 and *P*adj = 0.048, respectively).

**Figure 3:**
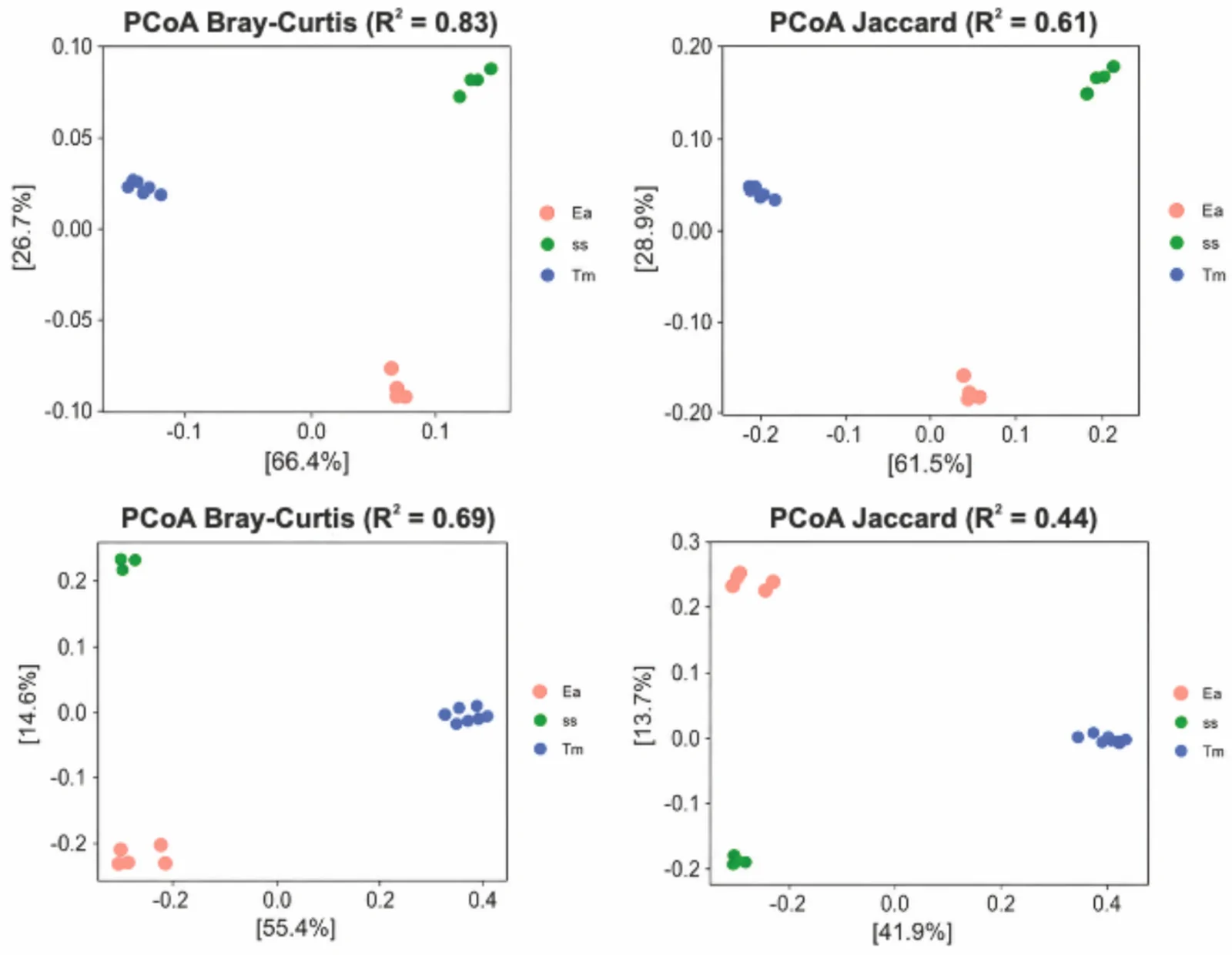
PCoAs of compositional beta-diversity estimates in bacteriomes (top) and mycobiomes (bottom) of sewage sludge (ss), *Eisenia andrei* casts (Ea), and *Tenebrio molitor* frass (Tm). The proportion of variation (R^2^) explained by treatment (ss, Ea, and Tm) in each plot is included.

PCoA of beta diversity estimates revealed clear segregation of all three treatment groups into tight, distinct clusters (Figure 3). This separation was consistent across all four-distance metrics (weighted UniFrac, unweighted UniFrac, Bray–Curtis, and Jaccard), and pairwise PERMANOVA confirmed significant differences in community structure between all group comparisons (all *P*adj ≤ 0.010; R^2^ = 0.71–0.87; Supplementary Table S3). These results confirm that gut transit by each invertebrate species drives the sludge bacteriome toward a distinct community state that is irreducible to a simple modification of the input.

### 3.2. Mycobiome Composition, Diversity, Structure, and Differential Abundance

Fungal community composition differed markedly among the three groups at both the phylum and genus levels (Figure 1 and Supplementary Table S1). At the phylum level, both ss and Ea were dominated by Basidiomycota (ss: 64.2%; Ea: 73.0% average) and Ascomycota (ss: 35.7%; Ea: 26.8% average), while Tm frass showed a marked shift toward increased Ascomycota dominance (45.7%) and reduced Basidiomycota (54.3%) relative to ss and Ea, consistent with the observed mycobiome collapse. At the genus level, the ss mycobiome was characterized by *Cutaneotrichosporon* (33.9 ± 1.3%) and *Apiotrichum* (14.6 ± 0.1%)—both members of the family Trichosporonaceae—as the dominant taxa, followed by Exophiala (3.2 ± 0.1%) and Cryptococcus (1.9 ± 0.3%), alongside a diverse assemblage of Ascomycota and Basidiomycota genera. The Ea mycobiome was similarly diverse, dominated by unclassified *Tremellomycetes* (29.0 ± 1.4%), *Apiotrichum* (25.3 ± 1.2%), Cutaneotrichosporon (6.8 ± 0.7%), and Aspergillus (5.4 ± 1.8%). The Tm mycobiome was severely reduced, with the community concentrated in a small number of taxa—unclassified Ascomycota (37.4 ± 7.8%), *Apiotrichum* (21.9 ± 5.8%), and *Cutaneotrichosporon* (21.7 ± 2.9%)—and very few additional taxa at detectable abundance.

Differential abundance analysis identified two fungal phyla showing significant differences between ss and Ea, three between ss and Tm and three between Tm and Ea (all *P*adj ≤ 0.046; Supplementary Table S4). At the genus level, 29 genera differed significantly between ss and Ea, 54 between ss and Tm, and 52 between Tm and Ea (all *P*adj ≤ 0.05; Supplementary Table S4).

Sewage sludge contained the highest fungal richness, followed by a slight reduction in Ea casts (−5%) and a collapse in Tm frass (−83%) (Figure 2). Shannon diversity declined from 3.859 ± 0.056 in ss to 3.171 ± 0.049 in Ea and 2.449 ± 0.065 in Tm. Fungal diversity declined significantly in Ea relative to ss for Shannon (*P*adj = 0.008) and inverse Simpson (*P*adj = 0.012), but ASV richness did not differ significantly between the two groups (*P*adj = 0.841), indicating that *E. andrei* processing suppressed evenness while largely preserving the number of detectable fungal taxa. *T. molitor* frass showed significant reductions across all three indices relative to ss (Observed *P*adj = 0.009; Shannon *P*adj = 0.004; Inverse Simpson *P*adj = 0.008). Ea casts and Tm frass differed significantly in both Observed richness and Shannon diversity (both *P*adj ≤ 0.009), confirming the near-complete mycobiome collapse in *T. molitor* frass.

PCoA of mycobiome beta diversity (Figure 3) showed clear segregation of all three groups for both Bray–Curtis and Jaccard distances, with pairwise PERMANOVA detecting significant differences across all comparisons (all *P*adj ≤ 0.011; R^2^ = 0.39–0.71; Supplementary Table S3). In both bacteriome and mycobiome, all three pairwise comparisons ss vs. Ea, ss vs. Tm, and Ea vs. Tm were individually significant, confirming that *T. molitor* frass and *E. andrei* casts differ from ss and from each other at the community level. Together, these results indicate that *E. andrei* gut transit selectively reorganizes the sewage sludge mycobiome while largely preserving fungal richness, whereas *T. molitor* gut transit produces a near-complete mycobiome collapse that sets the stage for the divergent pathogen dynamics described below.

### 3.3. Human Microbial Pathogen Differential Abundance

Having characterized the broader restructuring of the bacteriome and mycobiome imposed by each invertebrate, we next assessed human pathogen burden. We identified 29 HBP species across the 17 samples by cross-referencing the species-level taxonomy table with a curated HBP list [20]. The effects of the two invertebrate species on HBP dynamics diverged substantially, both relative to ss and from each other (Supplementary Figure S3 and Supplementary Table S5). The 29 HBPs collectively represented a small fraction of the total bacterial community, accounting for 0.12% of total reads in ss, 0.40% in Ea casts, and 3.58% in Tm frass, indicating that while HBP absolute abundance remained low in ss and Ea, *T. molitor* gut transit resulted in a marked relative enrichment of pathogenic taxa within the bacterial community.

Relative to ss, *E. andrei* processing produced a modest and largely balanced shift in the HBP community with only four HBPs showing significant differences, while *T. molitor* frass showed significant enrichment of 9 HBPs relative to Ea (Tm vs. Ea; *P*adj ≤ 0.05) in mean relative abundance (Supplementary Figure S3 and Supplementary Table S5). *Empedobacter falsenii* (231-fold more abundant; *P*adj < 10^−8^), *Vagococcus fluvialis* (84-fold more abundant; *P*adj ≤ 0.003), and *Turicibacter sanguinis* (12-fold more abundant; *P*adj = 0.020) were significantly enriched in Ea casts, while *Afipia clevelandensis* (17-fold less abundant; *P*adj < 10^−4^) was significantly reduced, with no enrichment of high priority clinical pathogens. *T. molitor* gut transit produced a markedly different and more concerning profile, with seven HBP species were significantly enriched in Tm frass relative to ss: *Vagococcus fluvialis* (1430-fold; *P*adj < 10^−8^), *Mammaliicoccus sciuri* (163-fold; *P*adj < 10^−9^), *Enterococcus faecium* (132-fold; *P*adj < 10^−9^), *Enterococcus avium* (101-fold; *P*adj < 10^−4^), *Morganella morganii* (83-fold; *P*adj < 10^−8^), *Providencia stuartii* (49-fold; *P*adj < 10^−5^), and *Turicibacter sanguinis* (11-fold; *P*adj < 10^−7^)—several of which are recognized opportunistic or nosocomial pathogens. *Collinsella aerofaciens* (29-fold less abundant; *P*adj < 10^−8^) and *Afipia clevelandensis* (14-fold less abundant; *P*adj < 10^−6^) were significantly depleted in Tm relative to ss.

Comparing the two invertebrate outputs directly (Supplementary Figure S3 and Supplementary Table S5), *Enterococcus faecium*, *Mammaliicoccus sciuri*, *Enterococcus avium*, *Vagococcus fluvialis*, *Morganella morganii*, and *Providencia stuartii* were substantially more abundant in Tm frass than in Ea casts, pointing to their selective amplification during *T. molitor* gut transit. Conversely, *Empedobacter falsenii* was enriched specifically in Ea casts relative to ss, indicating an earthworm-specific enrichment rather than a shared invertebrate effect. Together, these results indicate that while *E. andrei* processing leaves the HBP community broadly unaltered *T. molitor* gut transit selectively amplifies several clinically relevant bacterial pathogens.

We complemented the bacterial pathogen analysis by screening the mycobiome for human fungal pathogens (HFPs) against the WHO Fungal Priority Pathogens List [21] and VEuPathDB [22]. A total of 11 HFPs differed significantly between ss and Ea, two between ss and Tm, and 10 between Tm and Ea (all *P*adj ≤ 0.042; Supplementary Table S5). *Aspergillus caninus*, *Aspergillus chlamydosporus*, and multiple unclassified *Aspergillus* species were significantly more abundant in ss than in Ea casts (LFC = −8.2 to −10.6; *P*adj < 10^−3^) and were virtually absent from Tm frass. *Filobasidium uniguttulatum* a recognized opportunistic pathogen was significantly enriched in Ea casts relative to ss (LFC = +10.69; *P*adj < 10^−19^), while being near absent in Tm frass. In the Tm vs. Ea comparison, *Aspergillus caninus* (*P*adj = 0.007), *A. chlamydosporus* (*P*adj = 0.003), *Penicillium madriti* (*P*adj = 0.020), and *Cystobasidium slooffiae* (*P*adj = 0.004) were all significantly depleted in Tm relative to Ea. Together, the HFP data indicate that *E. andrei* processing selectively enriches *Cryptococcus* while depleting *Aspergillus*, whereas *T. molitor* gut transit results in neartotal elimination of detectable fungal pathogens because of the broader mycobiome collapse rather than targeted suppression.

### 3.4. Microbial Functional Diversity

Bacterial functional profiles were predicted from 16S rRNA data using PICRUSt2, with high predictive accuracy (mean weighted NSTI = 0.048 ± 0.001). The PCoAs (Supplementary Figure S4) of predicted functional properties showed a clear segregation of the ss samples from the earthworm cast and mealworm samples for the estimated distances (Bray–Curtis and Jaccard). Functional community structure differed significantly among all three substrate types across both distance metrics (PERMANOVA; Bray–Curtis: all *P*adj ≤ 0.007, R^2^ = 0.87–0.93; Jaccard: all *P*adj ≤ 0.008, R^2^ = 0.53–0.81; Supplementary Table S6).

We compared the functional potential (PICRUSt2) in the bacteriomes of ss, Ea and Tm across several selected metabolic categories (Supplementary Table S7 and Supplementary Figure S5). ARG abundance was significantly lower in both Ea casts and Tm frass relative to ss (ss vs. Ea: *P*adj = 5.07 × 10^−8^; ss vs. Tm: *P*adj = 3.45 × 10^−7^) and did not differ significantly between the two invertebrate outputs (*P*adj = 0.107). Furfural degradation potential was significantly higher in Ea than in both ss (*P*adj = 0.010) and Tm frass (*P*adj = 0.001), while ss and Tm did not differ (*P*adj = 0.574). Amino acid biosynthesis gene abundances differed significantly between ss and Ea (*P*adj = 0.003) and between Ea and Tm (*P*adj = 3.69 × 10^−4^), but not between ss and Tm (*P*adj = 0.382). Nitrogen metabolism gene abundances were significantly elevated in Tm frass relative to ss (*P*adj = 0.002) but did not differ between ss and Ea (*P*adj = 0.249) or between Ea and Tm (*P*adj = 0.216). Bisphenol A degradation potential was significantly lower in Tm relative to both ss (*P*adj = 0.039) and Ea (*P*adj = 4.26 × 10^−4^), while ss and Ea did not differ (*P*adj = 0.796). No significant differences were detected among groups for antibiotic biosynthesis or plant hormone synthesis pathways (all *P*adj > 0.05).

We extended the functional analysis to the mycobiome by assigning trophic modes and ecological guilds to fungal ASVs using FUNGuildR (Supplementary Table S8 and Supplementary Figure S6). A total of 38.1% of fungal ASVs across the dataset received guild assignments at Probable or Highly Probable confidence levels; these guild-assigned ASVs accounted for 12.6% of total fungal sequences. Among guild-assigned ASVs, 50.2% carried a trophic mode that included at least one Pathotroph component.

Trophic mode compositions (Supplementary Figure S6 and Supplementary Table S9) differed significantly among treatment groups for all seven trophic modes detected (Kruskal–Wallis, all *P*adj ≤ 0.007). The Saprotroph trophic mode was the most abundant across all groups and differed significantly among all three pairwise comparisons (ss vs. Ea: *P*adj = 0.004; ss vs. Tm: *P*adj = 4.46 × 10^−50^; Ea vs. Tm: *P*adj < 10^−70^), with mean abundance markedly higher in Ea (mean = 13.33) than in either ss (mean = 6.84) or Tm (mean = 6.98). Pathotroph abundance was significantly higher in Ea casts (mean = 115.9) than in both ss (mean = 23.3; *P*adj = 2.43 × 10^−34^) and Tm (mean = 0.80; *P*adj = 2.03 × 10^−39^). The Pathotroph–Saprotroph and Pathotroph–Saprotroph–Symbiotroph trophic modes were significantly depleted in Tm relative to both ss and Ea (all *P*adj < 10^−5^), and both modes also differed significantly between ss and Ea (*P*adj = 0.873 and *P*adj = 4.68 × 10^−6^, respectively). Pathotroph trophic mode abundance was significantly higher in Ea casts than in ss (*P*adj = 2.43 × 10^−34^) and near absent in Tm (*P*adj = 2.03 × 10^−39^), while Fungal Parasite guild abundance was near absent in Tm relative to Ea (*P*adj = 1.08 × 10^−13^). The near-complete mycobiome collapse in T. molitor frass resulted in near-total elimination of detectable functional guilds across all trophic modes.

## 4. Discussion

This study shows that the distinct gut physiologies of *E. andrei* and *T. molitor* restructure sewage sludge microbiomes differently and that the latter selectively amplifies a suite of clinically relevant bacterial pathogens that *E. andrei* processing leaves largely intact. This asymmetry runs counter to a framework in which invertebrate bioconversion is treated as a generically beneficial treatment for sewage sludge microbiomes and instead points to a species-specific outcome with direct regulatory implications.

### 4.1. Bacterial Taxonomic and Functional Restructuring

The reduction in bacteriome alpha diversity observed in both Ea and Tm relative to ss confirms that invertebrate gut transit functions as a selective ecological filter in both species, compressing the broad heterogeneous community of the input substrate into a narrower assemblage shaped by gut specific conditions [5,26]. The similar magnitude of this compression, approximately a 39–40% reduction in ASV richness across both groups, suggests that the filtering effect is driven primarily by gut transit rather than physiological differences between earthworm and mealworm digestive systems. This process appears to determine which subset of the microbial community can survive and proliferate [27].

The dominance of *Aeromonas*, *Acinetobacter*, *Flavobacterium*, *Gordonia*, and *Pseudomonas* in Ea casts points to the earthworm gut’s mucus-rich, aerobic-to-microaerobic gradient as the principal selective force. These genera are aerobic or facultatively anaerobic heterotrophs well suited to the organic-rich, oxygen-variable conditions created by gut mucus secretions and transit-associated redox changes [28]. The concurrent enrichment of Actinomycetota genera *Gordonia*, *Mycobacterium*, and *Nocardioides* reflects the earthworm gut’s documented capacity to selectively retain lignocellulose degraders and secondary metabolite producers [29]. In *T. molitor* frass, by contrast, the dominance of Bacillota (*Enterococcus*, *Lactococcus*, *Kurthia*, *Vagococcus*) alongside Actinomycetota (*Glutamicibacter*, *Corynebacterium*) reflects the larval mealworm gut’s distinctly different selective environment: higher alkalinity in the midgut, shorter transit time, and a characteristic larval microbiome that strongly favors Bacillota members [5,26].

The genus-level profile of Tm frass dominated by *Enterococcus*, *Lactococcus*, *Kurthia*, and *Vagococcus* closely mirrors the larval gut microbiome of *T. molitor* characterized in feeding experiments on other substrates [30,31]. A previous metabarcoding survey of *T. molitor* under mass-rearing conditions also reported *Enterococcus* and *Lactococcus* among the most abundant genera across developmental stages, colonies, and body compartments, including frass [32]. This suggests that mealworm gut transit imposes its own microbial signature on the substrate rather than selecting from within the existing sludge community. Beta diversity analyses showed a clear and significant separation between sewage sludge and Ea casts and Tm frass for both bacterial and fungal communities across all distance metrics tested. This again confirms that vermicomposting and insect bioconversion actively redirect community trajectories toward invertebrate-specific states.

The significant enrichment of nitrogen metabolism KOs in Tm frass relative to both ss and Ea (*P*adj = 0.002) is particularly noteworthy. Bacillota genera including *Enterococcus* and *Lactococcus* that dominate Tm frass include species with nitrogen cycling capacity, and enhanced nitrogen cycling potential may increase ammonia volatilization upon soil application, a concern for both nutrient use efficiency and atmospheric nitrogen loading that should be evaluated in soil mesocosm experiments.

The specific enrichment of furfural degradation KOs in Ea casts relative to both other groups adds a functionally valuable dimension to the earthworm-processed product. Furfural and its derivatives are toxic byproducts of lignocellulose breakdown that accumulate in composted and anaerobically digested material; their microbial degradation reduces phytotoxicity and improves substrate quality for downstream agricultural use [33]. This suggests that *E. andrei* processing may improve the agronomic safety of sludge-derived amendments beyond its direct effects on pathogen burden.

The reduction in predicted ARG abundance in both invertebrate outputs constitutes one of the most important biosafety outcomes of this study. Sewage sludge is a recognized hotspot for ARG dissemination, and the attenuation of resistance gene reservoirs in processed materials is a prerequisite for their safe application as organic amendments [3]. Earthworm gut digestion has been shown to substantially reduce ARG abundance in sludge: metagenomic analysis by Cui et al. [34] demonstrated that earthworm gut passage significantly attenuated both cell-free and cell-associated ARGs in excess activated sludge by reshaping the bacterial community and eliminating the principal ARG-carrying taxa [34]. Similarly, Huang et al. [35] reported a 41.5% decrease in total ARG abundance accompanied by reductions in plasmid types during earthworm-mediated vermicomposting of dewatered sludge, along with a concurrent reduction in human pathogenic bacteria [35]. The convergent ARG reduction observed here in both *E. andrei* casts and *T. molitor* frass, despite their contrasting effects on pathogen burden, indicates that gut transit exerts a strong and consistent filtering effect on the resistome regardless of invertebrate species. This effect is likely driven by the selective elimination of ARG-carrying microbial taxa during gut passage, a mechanism demonstrated across multiple invertebrate bioconversion systems [34,35]. The reduction has critical implications for the environmental safety of invertebrate-processed sludge: decreasing the ARG reservoir in organic amendments lowers the risk of resistance gene dissemination into agricultural soils, food chains, and surrounding ecosystems upon land application, thereby supporting the biosafety rationale for invertebrate-mediated sludge valorization.

### 4.2. Fungal Taxonomic and Functional Restructuring

The mycobiome data reveal a further dimension of the divergence between the two invertebrate species. While *E. andrei* casts maintained a moderately rich and diverse fungal community broadly comparable to ss in ASV richness, *T. molitor* frass underwent a severe mycobiome collapse, retaining only a narrow consortium of Ascomycota and Basidiomycota genera. The 83% loss of fungal ASV richness in Tm frass suggests that the *T. molitor* gut environment is profoundly hostile to most fungi, potentially due to its alkaline gut pH, high enzymatic activity, or antifungal compounds produced by the larval gut epithelium or its resident microbiota [36].

The persistence of saprotrophic guilds across ss and Ea casts is consistent with the earthworm gut’s well-documented capacity to support decomposer fungi through selective passage and mucus provisioning [5]. The significant enrichment of fungal parasite abundance in Ea casts relative to ss indicates selective retention or enrichment of specific guild-assigned fungal taxa during earthworm gut transit. The near-complete elimination of guild-annotated fungi in Tm frass reflects the broader mycobiome collapse rather than targeted suppression of any specific functional group. Beyond taxonomic restructuring, the fungal functional data indicate that the two invertebrate species also reshape ecological guild composition in contrasting ways. *E. andrei* casts retained a diverse guild structure and exhibited higher abundances of saprotrophic and fungal parasitic taxa relative to sewage sludge, suggesting preservation of functional roles associated with decomposition and fungal community regulation. In contrast, the near-complete loss of guild-assigned fungi in *T. molitor* frass indicates a substantial reduction in fungal functional redundancy. Such reductions may limit the resilience of the fungal community and reduce the range of ecological functions available during subsequent decomposition and soil incorporation. A mycobiome composed almost exclusively of Apiotrichum and Cutaneotrichosporon spp.—both members of the family Trichosporonaceae with relatively limited enzymatic repertoires compared to a diverse fungal community—represents a radical loss of fungal functional redundancy that may impair the long-term agronomic quality and biological stability of mealworm-processed material [37].

### 4.3. Human Pathogen Dynamics

The bacterial pathogen data establish a crucial biosafety distinction between the two invertebrate species. The simultaneous enrichment of *Enterococcus faecium*, *Mammaliicoccus sciuri*, *Morganella morganii*, and *Providencia stuartii* in Tm frass may constitute a biosafety profile that, under existing regulatory frameworks for land application of organic amendments including EU Regulation 2019/1009 and the proposed criteria under the revised Sewage Sludge Directive, could raise concerns regarding direct environmental release without prior hygienization. *E. faecium* is a leading cause of nosocomial infection in Europe and a primary carrier of vancomycin resistance (VRE). *M. sciuri* is a known reservoir of the *mecA* gene, which confers methicillin resistance and can transfer horizontally to *Staphylococcus aureus*. *M. morganii* and *P. stuartii* are Gram-negative opportunists capable of harboring extended spectrum beta lactamases (ESBLs) and are associated with infections in immunocompromised patients [20]. Their enrichment in Tm frass suggests that the *T. molitor* gut creates conditions permissive for these species despite of presence of antimicrobial peptides [38], potentially through alkaline midgut pH, short retention time, and a Bacillota-*dominated* resident microbiome. Nonetheless, since we did not quantify the overall microbial load of our samples (e.g., via qPCR), changes in pathogen relative abundance should be interpreted with caution. A previous study of the microbiota of *T. molitor* under standard mass-rearing conditions also identified several opportunistic bacterial pathogens preferentially associated with frass, but different than those reported here [32]. As here, this study also concluded that *T. molitor* frass, irrespective of the diet or system in which it is produced, is a recurrent reservoir of opportunistic bacterial pathogens that warrants targeted biosecurity monitoring before downstream use.

By contrast, *E. andrei* processing produced a modest and largely balanced shift in the HFP community, with no enrichment of high-priority clinical pathogens. The selective enrichment of *Filobasidium uniguttulatum* in Ea casts, an opportunistic pathogen relevant in immunocompromised patients, warrants monitoring in vermicompost-derived soil amendments. The depletion of *Aspergillus* spp. in Ea casts is broadly compatible with safe agricultural use. The virtual absence of detectable fungal pathogens from Tm frass reflects non-selective mycobiome collapse rather than targeted pathogen suppression and could not be interpreted as a biosafety advantage given the concurrent enrichment of bacterial HBPs documented. This is confirmed by a previous study of Tm under standard mass-rearing conditions where pathogens of the fungal genera *Aspergillus* and *Mucor* were still reported after gut transit in standard-diet mealworm systems [32].

### 4.4. Implications for Vermicomposting

The substantial restructuring of the bacteriome and mycobiome of sewage sludge following *E. andrei* digestion is consistent with previous studies [5,28,39,40,41], which have shown that microbiome modifications depend heavily on the earthworm species [39] and type of sewage sludge selected [40,41]. Furthermore, the effects on microbiome composition and diversity continue or intensify during microbial succession in the final stages of vermicomposting [5,28]. Although no study has characterized the *T. molitor* frass when fed sewage sludge, other studies have shown that its bacteriome varies with diet [42] and the environment when fed the same diet [26].

Together, our findings also indicate that invertebrate bioconversion of sewage sludge is not a uniform or interchangeable process, but rather a species-dependent ecological transformation with direct consequences for biosafety, functional capacity, and agronomic value. The contrasting outcomes observed in this study, with *E. andrei* producing a broadly safe and functionally enriched end product and *T. molitor* yielding a pathogen-enriched and mycobiome-depleted material, highlight the importance of developing species-specific biosafety assessment and management frameworks. These results challenge the suitability of a uniform regulatory approach for all invertebrate-processed sewage sludge.

*E. andrei* emerges as a particularly promising candidate for integrated sludge valorization across several independent dimensions. At the community level, earthworm gut transit substantially preserved fungal ASV richness (−5%) while selectively reorganizing community composition, thereby maintaining a diverse and functionally redundant mycobiome associated with the long-term biological stability of the processed material [37]. At the pathogen level, *E. andrei* processing left the human bacterial pathogen community broadly comparable to the input sludge, with no enrichment of high-priority clinical species such as *E. faecium*, *M. sciuri*, or *M. morganii*. At the resistome level, a significant and consistent reduction in predicted ARG abundance was observed [34,35]. At the functional level, *E. andrei* casts were specifically enriched in furfural degradation capacity [33], a trait that reduces phytotoxic byproduct accumulation and improves the agronomic safety and soil compatibility of the processed material. Taken together, these properties position vermicompost derived from *E. andrei* as a biologically stabilized, agronomically valuable material with a comparatively favorable biosafety profile. This profile is broadly compatible with the microbiological criteria for organic amendments applicable under EU Regulation 2019/1009 and the revised Sewage Sludge Directive framework, provided that chemical contaminants such as heavy metals remain within legislated thresholds [5].

For *T. molitor*, the microbial data indicate that direct land application of frass derived from sewage sludge may carry an unacceptable biosafety risk in the absence of post-processing hygienization. The simultaneous enrichment of *E. faecium*, *M. sciuri*, *M. morganii*, and *P. stuartii* species with documented antimicrobial resistance potential and nosocomial relevance [20] may constitute a biosafety profile that would require remediation before the material could be safely incorporated into agricultural soils or used in proximity to food crops. EU Regulation 2021/1925 already mandates a reference heat treatment of 70 °C for 60 min for insect frass intended as fertilizer or soil improver. Recent work on *T. molitor* frass has demonstrated that this treatment can bring *E. coli* and *Salmonella* counts into compliance with EU microbiological criteria, though Enterococcaceae reduction remains a challenge that is particularly relevant given the enrichment of *E. faecium* documented here [43]. Coupling insect bioconversion with a validated hygienization step, therefore, represents a necessary, rather than merely a precautionary, component of any sustainable *T. molitor*-based sludge valorization pipeline. Such a two-stage strategy—bioconversion followed by targeted heat treatment—would enable the full realization of the economic and circular-economy advantages of mealworm processing. These include well-established gains in larval biomass yield, protein production, and substrate volume reduction, while ensuring compliance with microbiological safety requirements for the land application of organic amendments.

This study has several limitations that should be considered when interpreting the results. The experiment was conducted using sewage sludge from a single source, which may limit the generalizability of the observed microbial and functional responses across sludges with different physicochemical characteristics or treatment histories. The work was performed under controlled laboratory conditions, which do not fully capture the environmental variability and operational complexity of large-scale bioconversion systems. The relatively short experimental duration provides a snapshot of microbiome restructuring immediately following gut passage but does not address the long-term stability of microbial communities, pathogen persistence, or functional traits during storage and subsequent environmental application. Future studies incorporating multiple sludge sources, pilot-scale systems, and longer monitoring periods would help validate and extend these findings. Given the predictive nature of PICRUSt2, further studies should consider using shotgun metagenomics and/or quantitative PCR (qPCR) analysis of selective antimicrobial resistance genes to confirm our results. We did not quantify the overall microbial load of our samples (e.g., using qPCR); consequently, we cannot ascertain whether changes in pathogen relative abundance reflect the selective depletion or enrichment of particular pathogens, or whether they are instead associated with broader changes in total microbial biomass during processing.

## Data Availability

Sequence files and associated metadata and BioSample attributes have been deposited in the NCBI (PRJNA1479214 and PRJNA1479220).

## Supplementary Materials

The following supporting information can be downloaded at: https://www.mdpi.com/article/10.3390/biotech15030062/s1, Figure S1: Rarefaction curves of ASV richness of the bacteriomes (top) and mycobiomes (bottom) of sewage sludge (ss), Eisenia andrei (Ea) and Tenebrio molitor (Tm); Figure S2: UpSet plots of ASVs unique and shared among bacteriomes (top) and mycobiomes (bottom) of sewage sludge (ss), Eisenia andrei (Ea) and Tenebrio molitor (Tm); Figure S3: Heatmap of human bacterial pathogen (HBP) abundance in the bacteriomes of sewage sludge (ss), Eisenia andrei (Ea) and Tenebrio molitor (Tm); Figure S4: Functional beta diversity PCoA plots (Bray–Curtis and Jaccard) of PICRUSt2 functional profiles in the bacteriomes of sewage sludge (ss), Eisenia andrei (Ea) and Tenebrio molitor (Tm); Figure S5: Predicted functional profiles in the bacteriomes of sewage sludge (ss), Eisenia andrei (Ea) and Tenebrio molitor (Tm); Figure S6: Abundance of fungal trophic modes and ecological guilds (FUNGuildR) in the myco-biomes of sewage sludge (ss), Eisenia andrei (Ea) and Tenebrio molitor (Tm); Table S1: Relative mean abundances (mean ± SE, %) of the most abundant bacterial and fungal phyla and genera in the bacteriomes and mycobiomes of sewage sludge (ss), Eisenia andrei (Ea) and Tenebrio molitor (Tm); Table S2: Differentially abundant bacterial phyla and genera (DESeq2) in the bacteriomes of sewage sludge (ss), Eisenia andrei (Ea) and Tenebrio molitor (Tm); Table S3: PERMANOVA analyses of taxonomic beta diversity in the bacteriomes and mycobiomes of sewage sludge (ss), Eisenia andrei (Ea) and Tenebrio molitor (Tm); Table S4: Differentially abundant fungal phyla and genera (DESeq2) in the mycobiomes of sewage sludge (ss), Eisenia andrei (Ea) and Tenebrio molitor (Tm); Table S5: Differentially abundant bacterial human bacterial pathogen (HBP) and human fungal pathogen (HFP) genera (DESeq2) in the bacteriomes and mycobiomes of sewage sludge (ss), Eisenia andrei (Ea) and Tenebrio molitor (Tm); Table S6: PERMANOVA analyses of functional beta diversity in the bacteriomes of sewage sludge (ss), Eisenia andrei (Ea) and Tenebrio molitor (Tm); Table S7: Statistical comparisons of predicted functional pathway categories in the bacteriomes of sewage sludge (ss), Eisenia andrei (Ea) and Tenebrio molitor (Tm); Table S8: FUNGuildR confidence assignment (Probable or Highly Probable) of trophic mode and ecological guild to fungal ASVs; Table S9: Statistical comparisons (Kruskal–Wallis) of fungal trophic mode and ecological guild abundances in the mycobiomes of sewage sludge (ss), Eisenia andrei (Ea) and Tenebrio molitor (Tm).
