# Supplementary material for: Eisenia andrei and Tenebrio molitor Divergently Restructure the Bacteriome and Mycobiome of Sewage Sludge with Contrasting Biosafety Consequences
Source: BioTech (Basel). 2026 Aug 3;15(3):62. doi: 10.3390/biotech15030062 (PMC13510514; doi:10.3390/biotech15030062)
Supplement: Supplementary file 1 [file biotech-15-00062-s001.zip › Supplementary Figure S1.pdf]

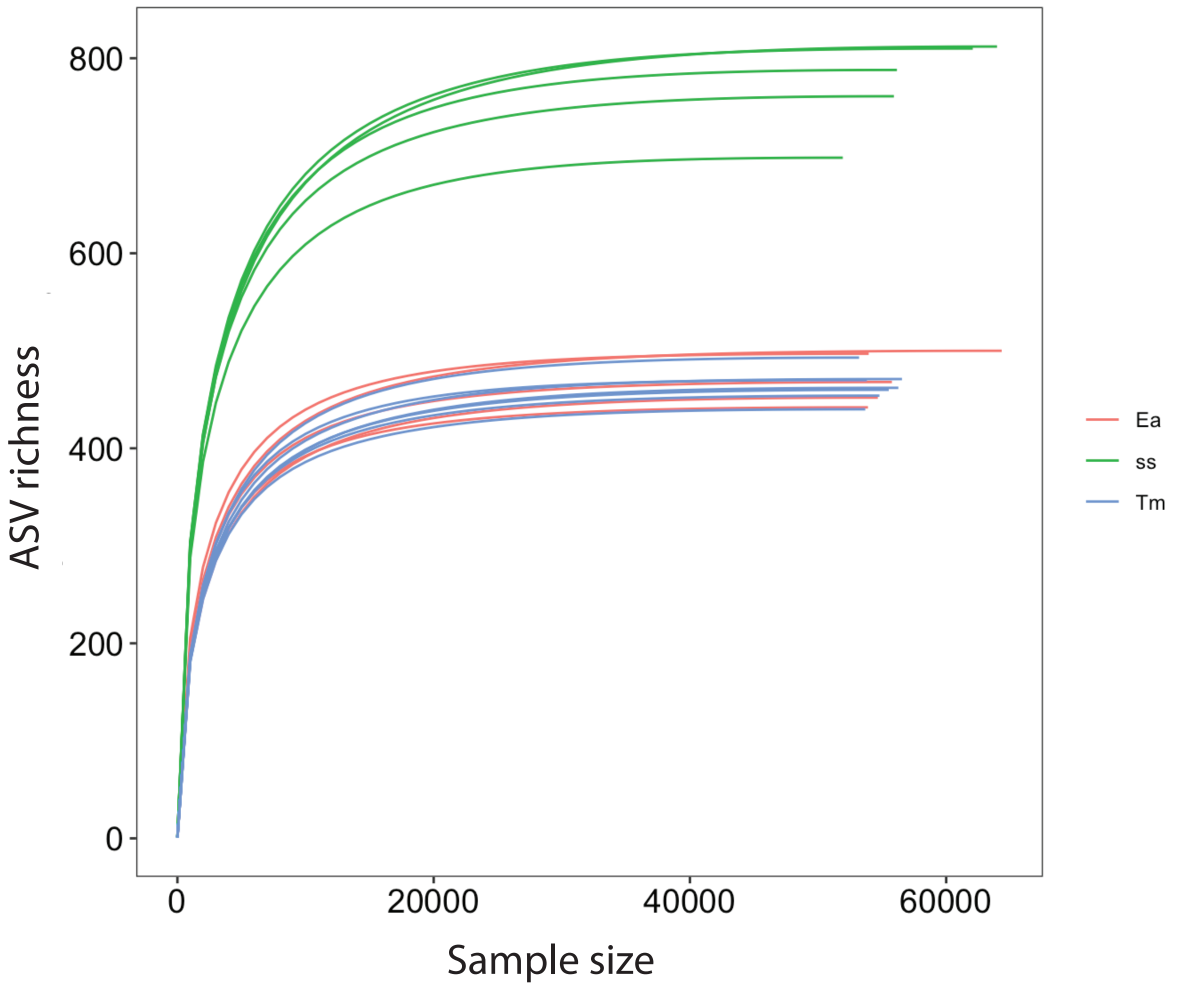
800
600
400
200
0
ASV richness
0
20000
40000
60000
Sample size
Ea
ss
Tm

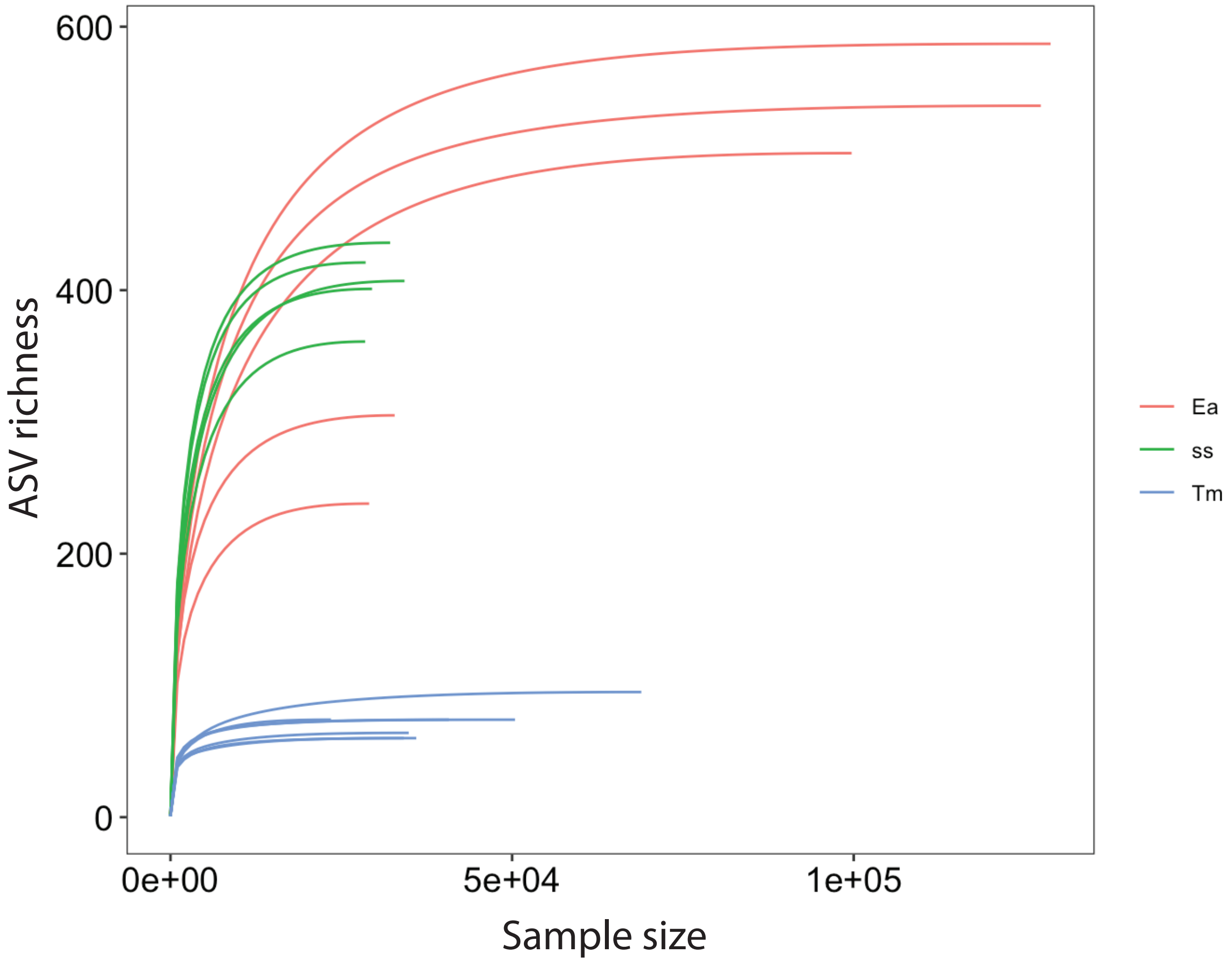
600
400
200
0
ASV richness
0e+00
5e+04
1e+05
Sample size
Ea
ss
Tm
