# Supplementary material for: Eisenia andrei and Tenebrio molitor Divergently Restructure the Bacteriome and Mycobiome of Sewage Sludge with Contrasting Biosafety Consequences
Source: BioTech (Basel). 2026 Aug 3;15(3):62. doi: 10.3390/biotech15030062 (PMC13510514; doi:10.3390/biotech15030062)
Supplement: Supplementary file 1 [file biotech-15-00062-s001.zip › Supplementary Figure S2.pdf]

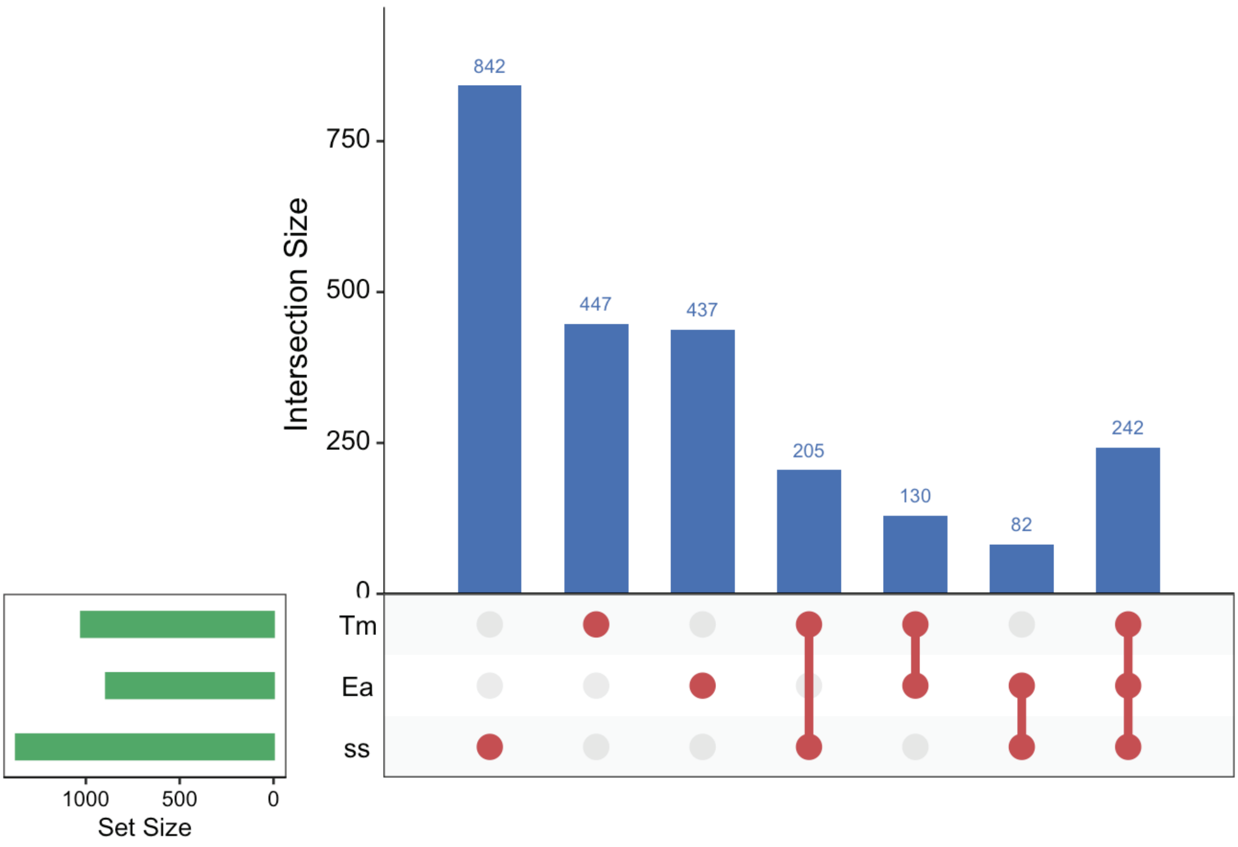

842
447
437
205
130
82
242
750
500
250
0
Intersection Size
Tm
Ea
ss
1000
500
0
Set Size

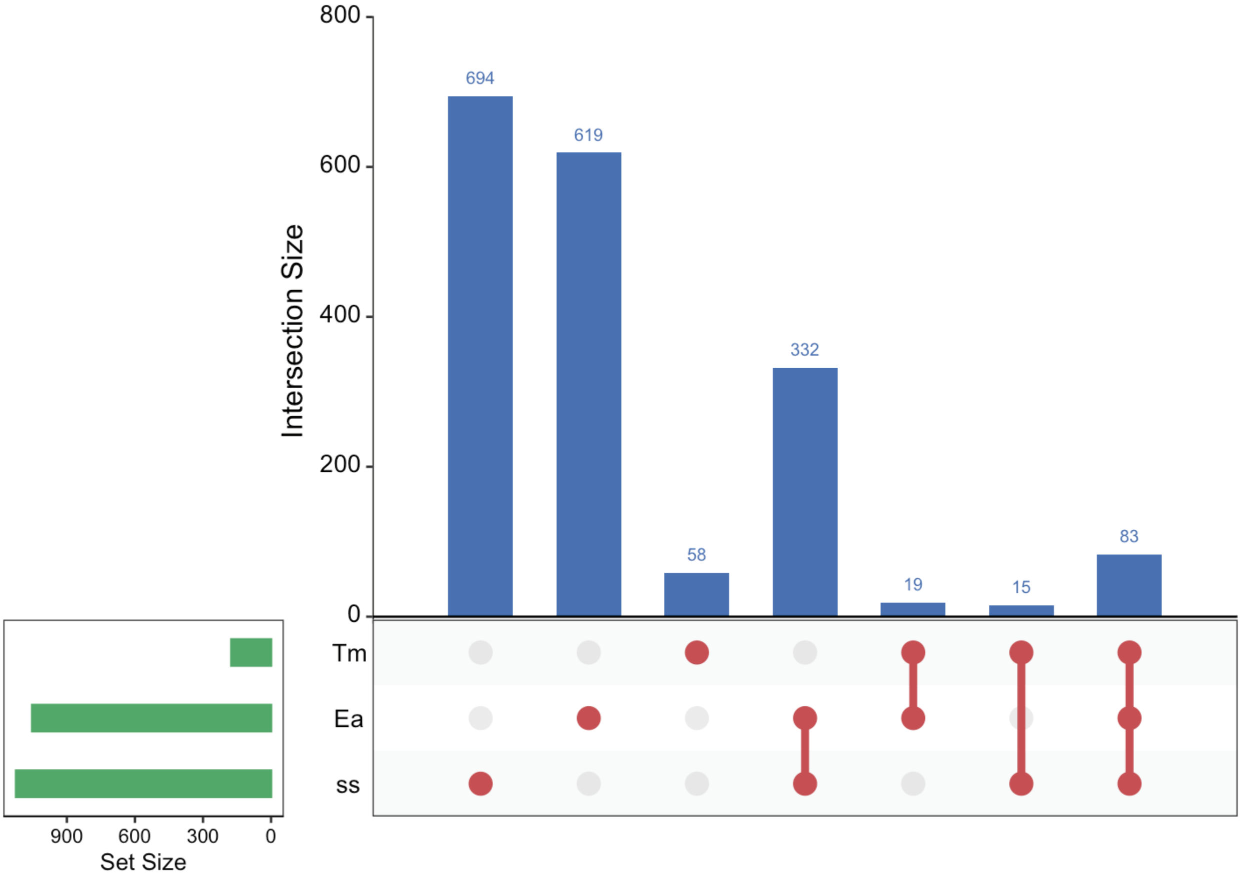

800
694
619
58
332
19
15
83
600
400
200
0
Intersection Size
Tm
Ea
ss
900
600
300
0
Set Size
