# Supplementary material for: Eisenia andrei and Tenebrio molitor Divergently Restructure the Bacteriome and Mycobiome of Sewage Sludge with Contrasting Biosafety Consequences
Source: BioTech (Basel). 2026 Aug 3;15(3):62. doi: 10.3390/biotech15030062 (PMC13510514; doi:10.3390/biotech15030062)
Supplement: Supplementary file 1 [file biotech-15-00062-s001.zip › Supplementary Figure S3.pdf]

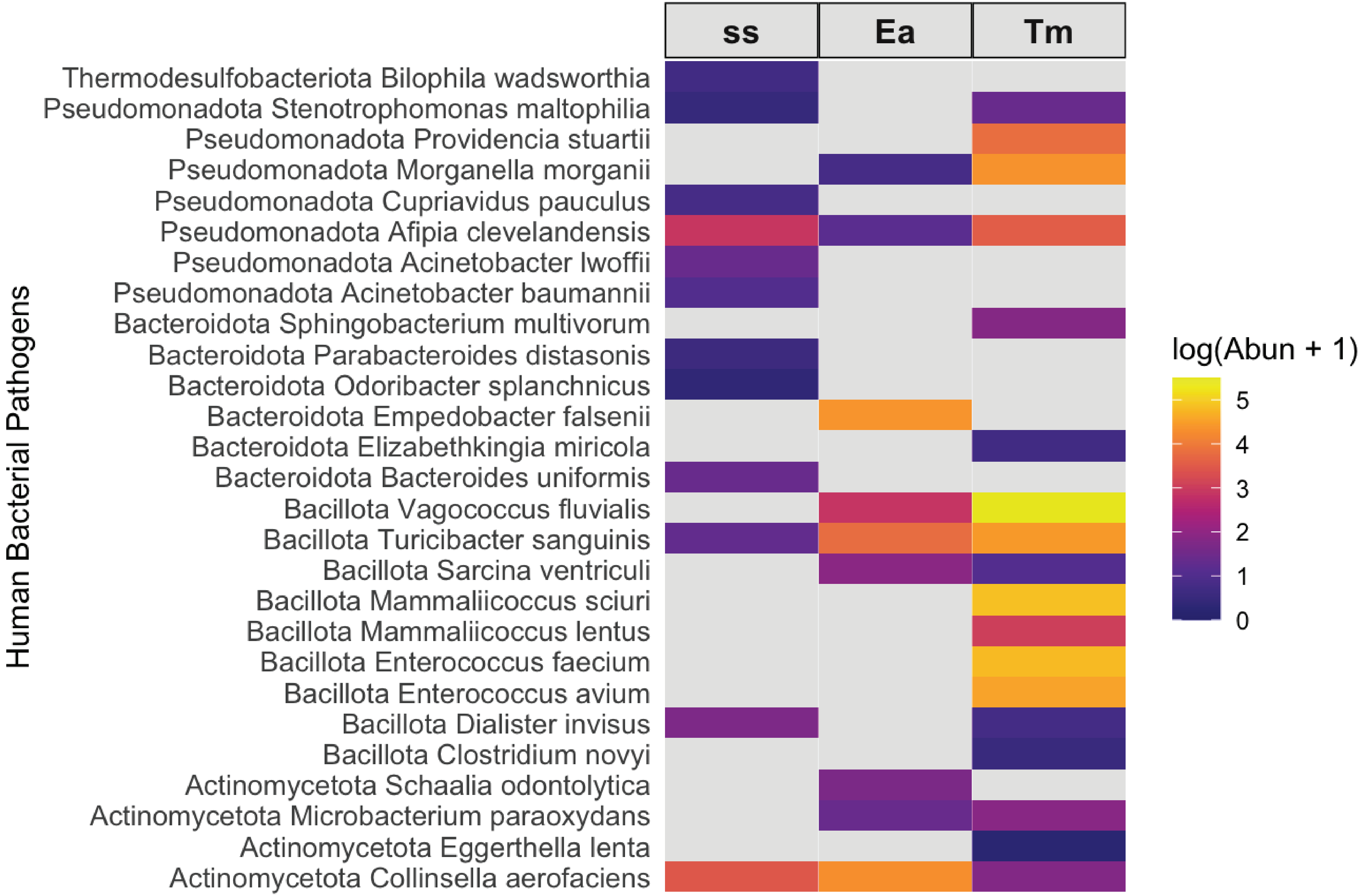
ss
Ea
Tm
Human Bacterial Pathogens
Thermodesulfobacteriota Bilophila wadsworthia
Pseudomonadota Stenotrophomonas maltophilia
Pseudomonadota Providencia stuartii
Pseudomonadota Morganella morganii
Pseudomonadota Cupriavidus pauculus
Pseudomonadota Afipia clevelandensis
Pseudomonadota Acinetobacter lwoffii
Pseudomonadota Acinetobacter baumannii
Bacteroidota Sphingobacterium multivorum
Bacteroidota Parabacteroides distasonis
Bacteroidota Odoribacter splanchnicus
Bacteroidota Empedobacter falsenii
Bacteroidota Elizabethkingia miricola
Bacteroidota Bacteroides uniformis
Bacillota Vagococcus fluvialis
Bacillota Turicibacter sanguinis
Bacillota Sarcina ventriculi
Bacillota Mammaliicoccus sciuri
Bacillota Mammaliicoccus lentus
Bacillota Enterococcus faecium
Bacillota Enterococcus avium
Bacillota Dialister invisus
Bacillota Clostridium novyi
Actinomycetota Schaalia odontolytica
Actinomycetota Microbacterium paraoxydans
Actinomycetota Eggerthella lenta
Actinomycetota Collinsella aerofaciens
log(Abun + 1)
5
4
3
2
1
0
