# Supplementary material for: Eisenia andrei and Tenebrio molitor Divergently Restructure the Bacteriome and Mycobiome of Sewage Sludge with Contrasting Biosafety Consequences
Source: BioTech (Basel). 2026 Aug 3;15(3):62. doi: 10.3390/biotech15030062 (PMC13510514; doi:10.3390/biotech15030062)
Supplement: Supplementary file 1 [file biotech-15-00062-s001.zip › Supplementary Figure S4.pdf]

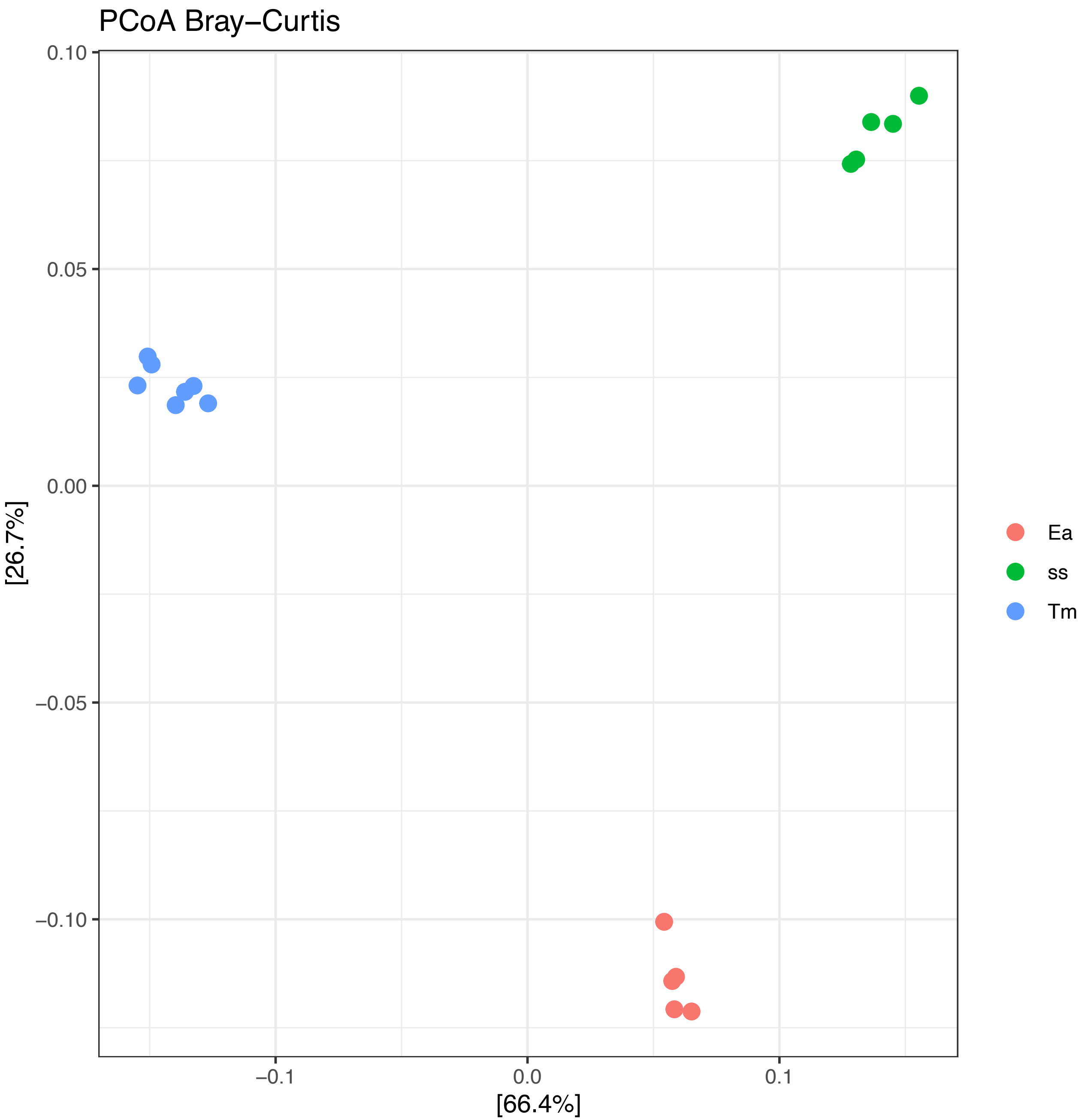
PCoA Bray−Curtis
0.10
0.05
0.00
−0.05
−0.10
[26.7%]
−0.1
0.0
0.1
[66.4%]
Ea
ss
Tm

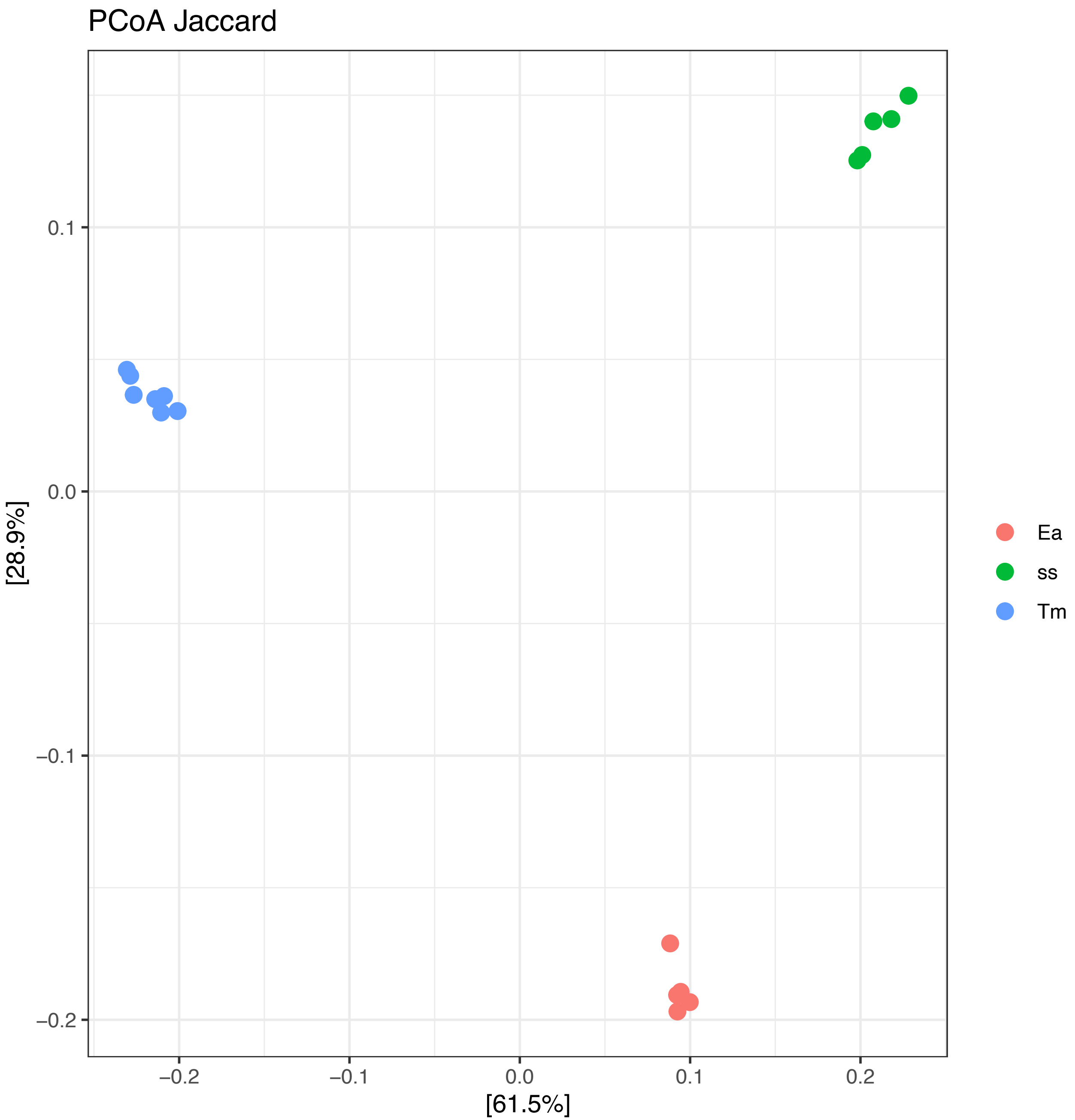
PCoA Jaccard
0.1
0.0
−0.1
−0.2
[28.9%]
−0.2
−0.1
0.0
0.1
0.2
[61.5%]
Ea
ss
Tm
