# Supplementary material for: Eisenia andrei and Tenebrio molitor Divergently Restructure the Bacteriome and Mycobiome of Sewage Sludge with Contrasting Biosafety Consequences
Source: BioTech (Basel). 2026 Aug 3;15(3):62. doi: 10.3390/biotech15030062 (PMC13510514; doi:10.3390/biotech15030062)
Supplement: Supplementary file 1 [file biotech-15-00062-s001.zip › Supplementary Figure S5.pdf]

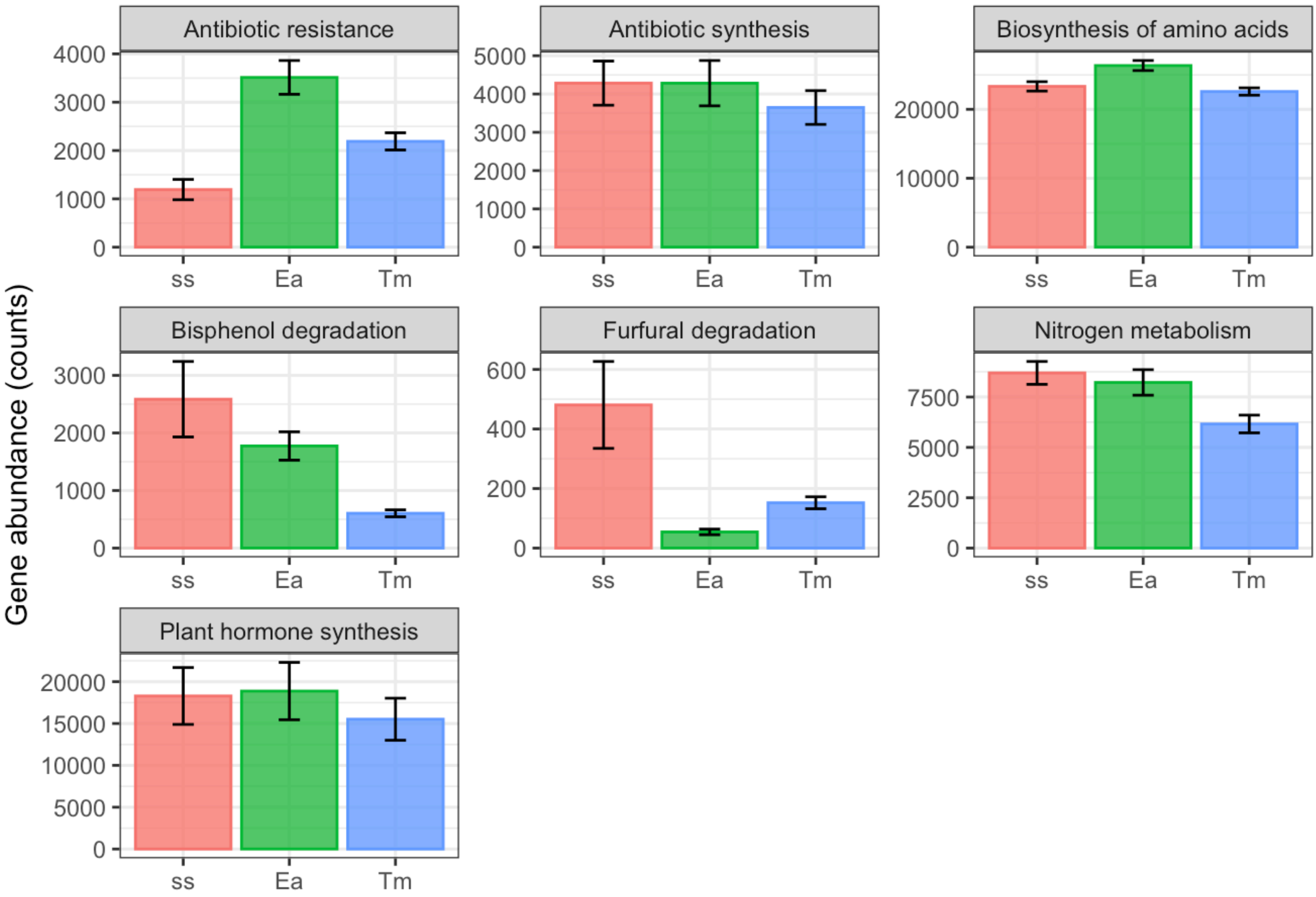
Gene abundance (counts)
Antibiotic resistance
Antibiotic synthesis
Biosynthesis of amino acids
Bisphenol degradation
Furfural degradation
Nitrogen metabolism
Plant hormone synthesis
ss
Ea
Tm
0
1000
2000
3000
4000
5000
10000
15000
20000
200
400
600
2500
7500
