# Supplementary material for: Eisenia andrei and Tenebrio molitor Divergently Restructure the Bacteriome and Mycobiome of Sewage Sludge with Contrasting Biosafety Consequences
Source: BioTech (Basel). 2026 Aug 3;15(3):62. doi: 10.3390/biotech15030062 (PMC13510514; doi:10.3390/biotech15030062)
Supplement: Supplementary file 1 [file biotech-15-00062-s001.zip › Supplementary Figure S6.pdf]

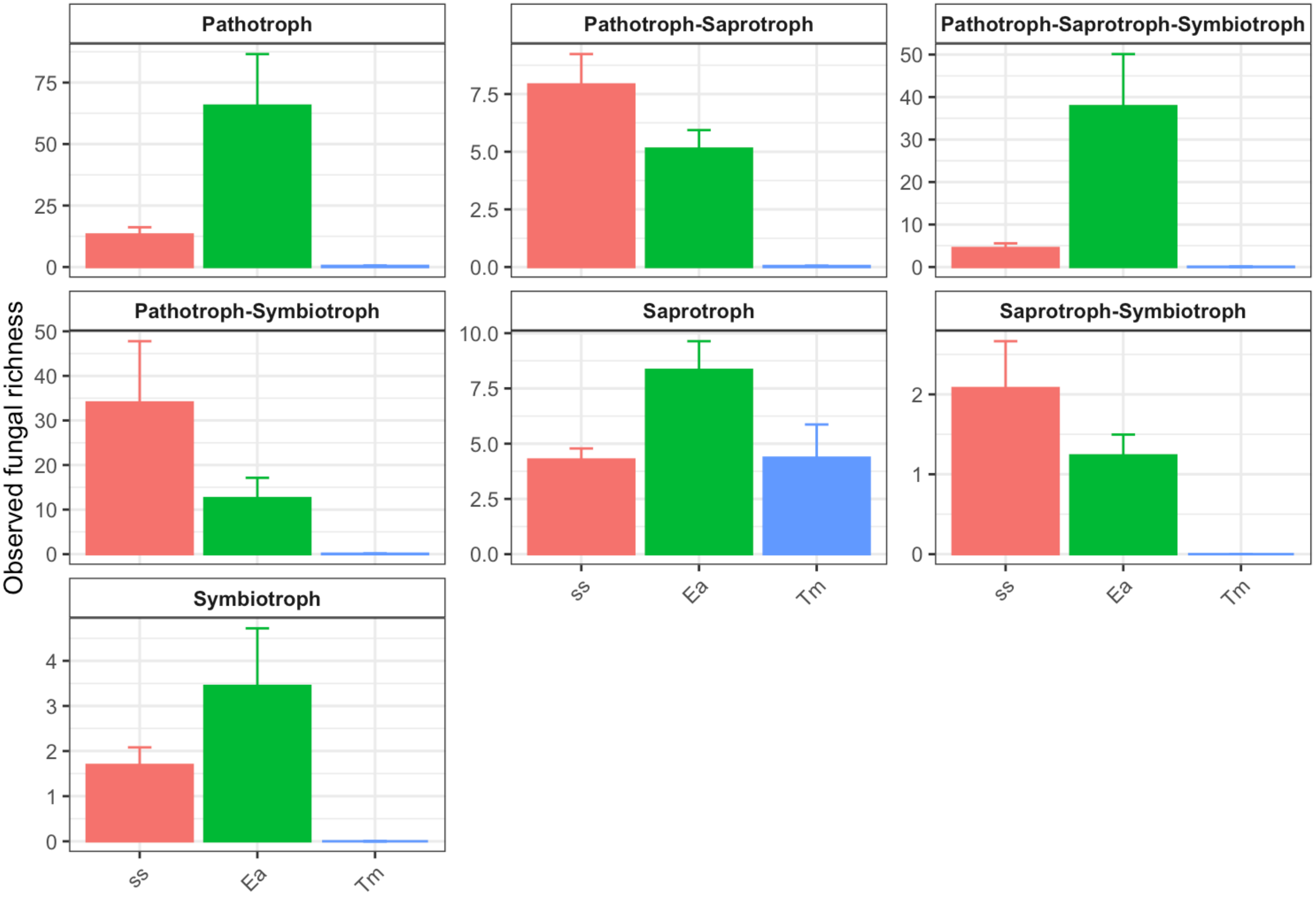
Pathotroph
Pathotroph-Saprotroph
Pathotroph-Saprotroph-Symbiotroph
Pathotroph-Symbiotroph
Saprotroph
Saprotroph-Symbiotroph
Symbiotroph
Observed fungal richness
75
50
25
0
7.5
5.0
2.5
0.0
50
40
30
20
10
10.0
2
1
4
3
ss
Ea
Tm
